# Contrasting Susceptibilities to Flavescence Dorée in *Vitis vinifera*, Rootstocks and Wild *Vitis* Species

**DOI:** 10.3389/fpls.2016.01762

**Published:** 2016-11-29

**Authors:** Sandrine Eveillard, Camille Jollard, Fabien Labroussaa, Dima Khalil, Mireille Perrin, Delphine Desqué, Pascal Salar, Frédérique Razan, Cyril Hévin, Louis Bordenave, Xavier Foissac, Jean E. Masson, Sylvie Malembic-Maher

**Affiliations:** ^1^UMR 1332 Biologie du Fruit et Pathologie, Institut National de la Recherche Agronomique, Université de BordeauxVillenave-d’Ornon, France; ^2^UMR 1131 Santé de la Vigne et Qualité du Vin, Institut National de la Recherche Agronomique, Université de StrasbourgColmar, France; ^3^UMR 1287 Ecophysiologie et Génomique Fonctionnelle de la Vigne, Institut National de la Recherche Agronomique, Université de BordeauxVillenave-d’Ornon, France

**Keywords:** genetic traits, grapevine, phytoplasma, *Scaphoideus titanus*, symptoms, transmission

## Abstract

Flavescence dorée (FD) is a quarantine disease of grapevine, involving interactions between the plants, leafhopper vectors, and FD phytoplasma. Characterizing the susceptibility of vine varieties could limit disease propagation. After extensive surveys in vineyards, we showed that Cabernet Sauvignon (CS) is highly susceptible, with a high proportion of symptomatic branches and phytoplasma titers, in contrast to Merlot (M). Localized insect transmissions and grafting showed that phytoplasma circulate in the whole plant in the CS cultivar, but in M they are restricted to the transmission point. Insect-mediated transmission under high confinement mimicking natural conditions confirmed these phenotypes and allowed the classification of 28 *Vitis* accessions into three distinct categories, according to the percentage of infected plants and their phytoplasma titers. Reduced symptoms, low phytoplasma titers, and low percentages of infected plants were found to be associated in the *Vitis vinifera* cultivars tested. Interestingly, the low susceptibility of M was observed for one of its parents, i.e., Magdeleine Noire des Charentes. Rootstocks and their *Vitis* parents, although having high percentages of infected plants and intermediate to high phytoplasma titers, shared a symptomless response. This is troubling, because rootstocks can constitute a silent reservoir of contamination in mother plants or when they grow wild nearby vineyards. Altogether, data suggest distribution of genetic traits within the *Vitis* genus involved in insect-mediated phytoplasma transmission, multiplication, circulation, and symptom development.

## Introduction

Phytoplasmas are non-cultivated wall-less bacteria belonging to the class Mollicutes (Weisburg et al., 1989) and are responsible for hundreds of diseases in ornamentals, cultivated plants, and weeds, worldwide. Restricted to the phloem sieve tubes, they are transmitted from plant to plant by phloem sap-sucking leafhoppers, and through grafting (Weintraub and Beanland, 2006). The Flavescence dorée (FD) phytoplasma (FDp) is associated with one of the most important and destructive epidemic diseases of grapevine, which has become a major threat to European viticulture (Boudon-Padieu, 2002; Chuche and Thiéry, 2014). Symptoms are leaf yellowing or reddening, with downward rolling, incomplete lignification of canes, abortion of flowers, and grape wilting (Caudwell, 1957). It can reduce grape harvests significantly and greatly weaken the vineyards development (Pavan et al., 2012). FDp is transmitted by *Scaphoideus titanus* Ball (Schvester et al., 1963), a leafhopper living and feeding on *Vitis* that was accidentally introduced into Europe from North America, one century ago (Papura et al., 2012). Recent studies report that FD phytoplasma originated from European wild alders and clematis that surround vineyards (Angelini et al., 2004; Arnaud et al., 2007; Filippin et al., 2009). With respect to Europe’s situation, this suggests a recent association with *Vitis vinifera* that occurred when *S. titanus* invaded South European vineyards, and thus propagated the phytoplasma on grapevines. Due to its high potential for causing epidemic disease in *V. vinifera*, FD is listed in Quarantine Pests for Europe, published by the European Union.

Infected, symptomatic plants cannot be cured from phytoplasma. The only way to control the disease is through plant destruction, insecticide treatments against the vector, and production of disease-free material for planting. Despite these mandatory methods, numerous outbreaks have been reported in Europe. First described in Southwestern France, FD expanded in France and spread to other European countries, from Portugal to Hungary (Foissac and Maixner, 2013; EFSA Panel on Plant Health (PLH), 2014). However, the prophylactic control has deleterious economic, social, and environmental impacts, so better adapted, and environmentally friendly strategies are awaited to control FD.

The disease propagation depends on the inoculum abundance, because there is a correlation between FDp titers in the plant and acquisition efficiency by *S. titanus* (Galetto et al., 2014). However, it also depends on insect population sizes (Morone et al., 2007), as well as on transmission efficiency. One possible strategy for controlling the disease would be the planting of grapevine rootstocks and cultivars that are less susceptible to FDp transmission and/or FDp multiplication. However, phenotypic and genetic evidence for such traits are lacking. Field observations, and a few experimental inoculations of FDp, have shown that non-grafted rootstocks displayed either attenuated, or no symptoms (Moutous et al., 1977; Caudwell et al., 1994a,b; Boudon-Padieu, 1996; Borgo et al., 2009). Nevertheless, some of the accessions were shown to be infected by the phytoplasma, as FDp could be transmitted through grafting to *V. vinifera* cultivars (Caudwell et al., 1994a; Borgo et al., 2009). Today, data concerning the susceptibility of the main rootstock varieties are too scarce. In *V. vinifera*, evidence for resistance has never been reported, but differences in susceptibility between cultivars have been observed in vineyards on the basis of disease incidence, symptom severity, as well as plant’s ability to recover (Boudon-Padieu, 1996). One study performed in vineyards showed that the cultivar Barbera, in contrast to Nebbiolo, was highly susceptible to FD, with high pathogen titers (Roggia et al., 2014). Such contrasting data question the relationship between symptom development and FDp titers. Due to the mandatory uprooting of FDp contaminated stocks, field studies are based on low numbers of plants, precluding solid conclusions.

We characterized the susceptibility of *V. vinifera* to FD disease by measuring the percentage of infected plants, the symptom severity, and the FDp titers in hundreds Cabernet Sauvignon (CS) and Merlot (M) plants growing in vineyards. We developed a controlled FDp transmission assay by *S. titanus* under confined greenhouse conditions which allowed refining field conclusions for CS and M phenotypes. This protocol also allowed the characterization of the response to FD in 28 different *V. vinifera* cultivars, rootstocks and wild *Vitis* sp. Data suggest the segregation of genetic traits within this germplasm and open for the first time, the possibility of breeding for FD resistance.

## Materials and Methods

### Phytoplasma Strain

FDp strain FD-PEY05 was used for inoculations. It was transmitted to broad bean (*Vicia faba*) by *S. titanus* leafhoppers, collected in 2005 in FD-infected vineyards in Peyrière, South-west France (Papura et al., 2009). The FD-PEY05 genotype belongs to the map-FD2 genetic cluster, and is widely distributed in the main outbreaks of Western Europe (Arnaud et al., 2007; Papura et al., 2009). The strain has been maintained since then by serial transmission on broad bean, using *Euscelidius variegatus* as a vector (Caudwell et al., 1972).

### Plant Material

Details of *V. vinifera* cultivars, rootstock hybrids, and wild *Vitis* species used in this study are described in **Table 1**. Green growing arms of accessions were sterilized and introduced *in vitro* according to Perrin et al. (2004). For wild species, the concentration of calcium hypochlorite was reduced (2% final active chloride). Accessions were maintained by vegetative propagation on WPM medium (Lloyd and McCown, 1980), supplemented with 15 g.l^-1^ sucrose and solidified with 6.5 g.l^-1^ Difco bacto agar.

**Table 1:** *Vitis vinifera* cultivars, rootstocks, and wild *Vitis* species used in the study were collected in the repository of INRA Bordeaux, ^∗^Colmar, and ^∗∗^vignoble des Charentes.

Name	ID number or clone	Abbreviation
Cabernet Franc N	312	CF
Cabernet Sauvignon N	15 and 337	CS
Chardonnay B^∗^	76	CHAR
Grenache N^∗^	136	GR
Magdeleine Noire des Charentes^∗∗^		MAGD
Merlot N	181	M
Pinot Noir N^∗^	162	PN
Sauvignon B	316	SAU
Syrah N^∗^	174	SY
110 Richter	151	110R
3309 Couderc	144	3309
41 B Millardet et de Grasset^∗^	172	41B
Kober 5 BB^∗^		5BB
Nemadex Alain Bouquet	1163	NEM
Riparia Gloire de Montpellier	142	RGM
Sélection Oppenheim 4	762	SO4
*Vitis amurensis* *V. berlandieri* *Vitis champinii* *V. coignetiae* *V. doaniana* *V. labrusca* *Vitis longii*	10151 10585 10164 2252 10165 10308 10942	ARM BER CHAM COI DO LAB LON
*V. pentagona*	R4-n°70-73	PEN
*V. rubra*	10919	RUB
*V. rupestris*	10334	RUP
*V. simpsonii*	10968	SIM
*V. vinifera* subsp. *sylvestris*	Chalonnes (Charentes)	SYL

*Parentage of the following accessions: CS (CF × SAU); CHAR (PN × Gouais B); M (MAGD × CF); SAU (Savagnin × ?); SY (Dureza N × Mondeuse Blanche; Boursiquot et al., 2009; Lacombe et al., 2013); 110R (V. berlandieri cv. Rességuier n°2 × V. rupestris cv. Martin); 3309 [V. riparia (tomenteux) × V. rupestris cv. Martin]; 41B (V. vinifera cv. Chasselas B × V. berlandieri); 5BB (V. berlandieri × V. riparia Euryale Rességuier); NEM {VMH 8771 [(Cabernet Sauvignon × Alicante Henri Bouschet) × Muscadinia rotundifolia NC 184-4] × 140 Ruggeri (V. berlandieri cv Rességuier n°2 × V. riparia Euryale Rességuier)}; RGM (V. riparia Michaux); SO4 (V. berlandieri × V. riparia Euryale Rességuier).*

All graftings were done with *in vitro* grown plantlets. The rootstock consisted of a one-bud stem segment of CS or M (ca. 10 mm), onto which, a one bud-stem segment of Chardonnay scion was grafted (“English groove graft”). Plant vessels from the two parts joined within 4–6 days (data not shown), and plantlets were grown until the four to five internode developmental stage.

**FIGURE 1:**
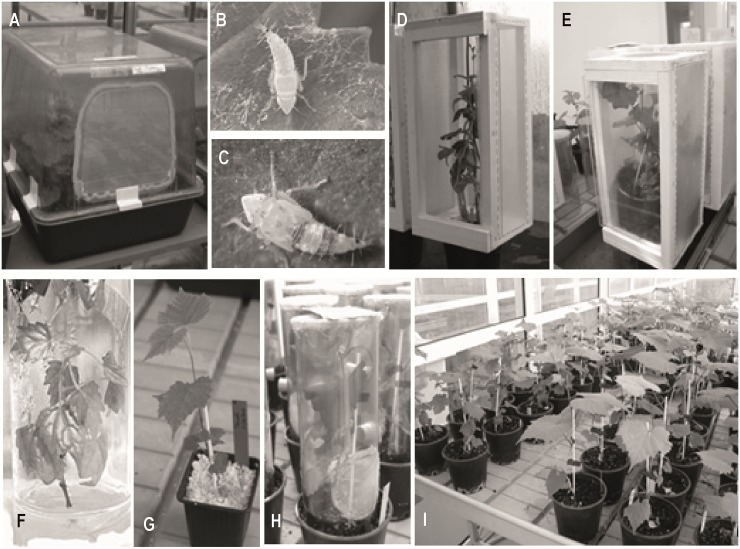
**A 24- to 32-week protocol to reproducibly obtain vines plants infected by *Scaphoideus titanus* under high confinement greenhouse conditions.** Hatching of *S. titanus* from grapevine canes collected in the vineyard, 6–8 weeks **(A–C)**. FDp acquisition by L3–L5 *S. titanus* larvae on FDp-infected broad bean, 1 week **(D)**. Latency period on grapevine, 3–4 weeks **(E)**. Acclimatization of grapevine *in vitro* plantlets, 8 weeks **(F,G)**. FDp transmission to grape plants by infectious *S. titanus*, 1 week **(H)**. Incubation of plants, 5–10 weeks, until sampling for FDp quantification and symptoms evaluation **(I)**.

All *in vitro* plantlets (four to five internodes) were transferred for acclimatization in perlite-containing pots (**Figures 1F,G**). After 1 month of progressive weaning, plantlets were planted into 12 cm pots, filled with soil (Klassman n°5) for further development. Plants were grown at 25 ± 2°C, and 50–80% humidity, under illumination of 50 μEm^-2^.s^-1^ for a 16 h period (Osram, Lumilux), and under 400 W high pressure sodium lamps for a 14 h period, *in vitro* and in the greenhouse, respectively.

#### Infectious Vector for Transmission Assays

The protocol for obtaining infectious *S. titanus* is illustrated in **Figures 1A–E**. The *S. titanus* eggs were obtained from 2-year-old *V. vinifera* canes, collected in January, from FD and insecticide-free vineyards from Burgundy. Canes were stored at 4–8°C in a cold chamber. Hatchings were performed as described in Chuche et al. (2015) by incubating the canes at 22°C with daily water sprayings. The first eggs hatched 3 weeks later and L3–L5 larvae were transferred by groups of 100 onto FD-PEY05 infected broad beans for FDp acquisition. One week later, insects were transferred onto CS cuttings for a latency period of 3–4 weeks. For the experiment of FDp diffusion in M and CS plants, latency period was done on rootstock 41B.

#### Transmission and Sampling in Confined Conditions

After the latency period, seven infectious *S. titanus* were collected randomly and encaged on each whole plant for FDp transmission during 7 days (**Figure 1H**). At the end of the 1 week transmission period, *S. titanus* were collected, survival rates were recorded, and 80–100 randomly selected individuals were kept frozen for further phytoplasma quantification. Between 9 and 20 plants, 20–30 cm high, were inoculated for each *Vitis* genotype. For each inoculation experiment, three to six *Vitis* genotypes could be tested and CS plants were included as the susceptible reference and positive controls. All plants were then incubated at 25°C constant, which is the optimum temperature for the multiplication of FDp (Salar et al., 2013; **Figure 1I**). At 5 and 10 weeks post-transmission (wpt), the proportion of infected plants was calculated and symptoms were evaluated. Then, all midribs, petioles, and stems of each plant or plant sections were cut into pieces and stored at -20°C for FDp quantification.

For the experiment of FDp diffusion in M and CS plants, seven infectious insects were encaged on the 3rd or the 4th leaf of the apical part only. At 10 wpt, 10 CS and 10 M were cut in different sections (**Figure 6A**): number 1 is the base of the plant, number 2 includes the transmission leaf, and 3 is the apex. Sections named R are the lateral branches grown at the base of the plant.

For grafted *in vitro* plants, seven infectious insects were encaged on the grafted scions (Chardonnay) solely. Scions (Chardonnay) and rootstocks (CS or M) were sampled separately 15 wpt and stored at -20°C for FDp quantification.

### Observation and Sampling in Vineyard

**FIGURE 2:**
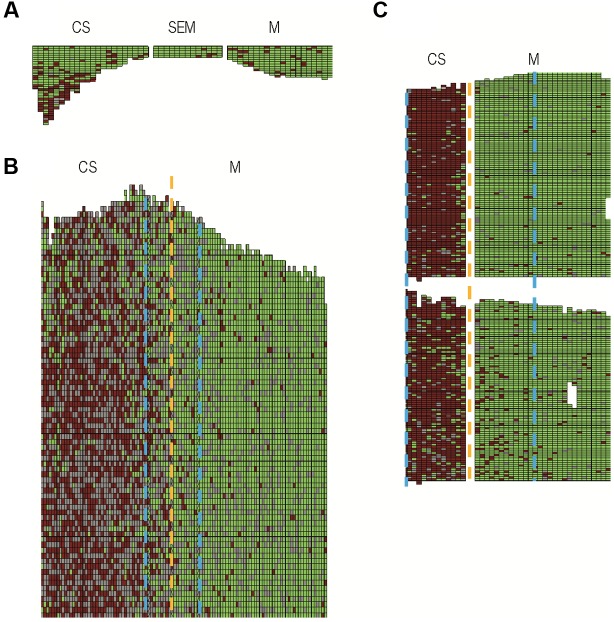
**Distribution of asymptomatic, symptomatic, and missing vine stocks of Cabernet Sauvignon and Merlot, in the plots of Beychac, Baurech, and Ambès (A–C)** (green, red, and gray boxes, respectively). CS, Cabernet Sauvignon; M, Merlot; SEM, Semillon. Rootstocks were 3309C and SO4 at Baurech and Ambès, respectively. At Beychac, CS was grafted onto 101-14 and M onto 420A or 110R. Dashed yellow and blue lines indicate the limits between CS and M plots and the 10–12 rows-large band on both sides of this limit, respectively.

Three vineyards plots from Bordeaux area (Sites of Ambès, Baurech, and Beychac) were selected for growing side by side CS and M of the same age (except in Beychac), on the same rootstock (except in Beychac) and with important FD disease outbreaks (**Figure 2**). The mapping of the symptomatic plants was performed and symptom’s severity was recorded at the end of September 2010, and 2014 for the sites of Baurech and Ambès respectively and in mid-June 2011 for the site of Beychac. Stocks were classified in four categories: >0–25%, 26–50%, 51–75%, and >75% symptomatic branches on the plant. Twenty-eight to 35 symptomatic plants of each cultivar, distributed in the four categories, were randomly selected in the plot and 8–10 petioles from symptomatic leaves were sampled on the symptomatic branches at the end of September in Baurech and Ambès and at mid-June, mid-July, and mid-August in Beychac. Petioles were also sampled on the non-symptomatic parts of each stock and also on non-symptomatic stocks as controls.

After uprooting of vine plots, it is quite frequent to observe regrowth of rootstocks from undestroyed roots after years, and those invade neighboring canopies. Such wild rootstocks can also result from regrowth of cane cuttings which were thrown in the environment when, in the old times, grafting was directly performed in the plots. Such wild rootstocks regrowth, growing near FD-infected plots, were sampled at the end of the summer 2010 and 2012. They were mainly *Vitis riparia*, 161–49 *V. riparia* × *Vitis berlandieri*, and Clinton *V. riparia* × *Vitis labrusca*. Some symptomatic *V. vinifera* cultivars (CS, Semillon, Sauvignon) were also sampled in the neighboring FD-infected plots.

After washing, 0.5 g petioles were homogenized and frozen for further nucleic acid extraction and FDp quantification.

### Nucleic Acids Extraction

It was done as described in Maixner (2005), with a ratio of 0.5 g fresh weight (gFW) ground in 3 ml cetyltrimethylammonium bromide (CTAB) buffer. For the extraction procedure, 1 ml of the ground mix was used, and the nucleic acids were re-suspended in 80 μl TE 1× (Tris 10 mM, ethylenediaminetetraacetic acid (EDTA) 1 mM pH 8). For nucleic acid extraction from insects, a single individual was ground in 250 μl CTAB buffer, and the final pellet was re-suspended in 40 μl of TE 1×. Healthy grapevines were used as negative controls in each extraction series. Nucleic acid concentrations were determined with a NanoVue^TM^ Plus (GE Healthcare) and reduced to 40 ng.μl^-1^ for qPCR analysis. Purity was assessed by calculating the ratio of the absorbance at 260 nm over the absorbance at 280 nm and at 230 nm.

### FDp Detection, Quantification, and Genotyping

FDp cells were detected and quantified in plants and in insects, by N′,N′-dimethyl-N-[4-[(E)-(3-methyl-1,3-benzothiazol-2-ylidene)methyl]-1-phenylquinolin-1-ium-2-yl]-N-propylpropane-1,3-diamine (SYBR) Green absolute quantitative real-time PCR of the *tuf* gene, as it is present as a unique copy in the bacterial chromosome (Malembic-Maher et al., 2008). Determined primers 3Fbl (5′-TGAAGATCCAGTACGTGATTTAGAC-3′) and 3Rl (5′-TTTTAGTTTCTTTAATACCTATGATTTC-3′) are positioned at 456–480 and 613–586, respectively on the FD92 *tuf* sequence FN561880 and led to the amplification of a 158 bp fragment. The conditions and validation of the PCR on the total nucleic acid extracts from plants and insects are detailed in the Supplementary Material (Supplementary Figure S4). The FDp strains detected in field samples were genotyped by sequencing the *map* gene (Arnaud et al., 2007).

### Data Analysis

Box plots, histograms and curves representing FDp titers were obtained using Microsoft Excel, with calculations of the mean FDp titers (number of FDp cells.gFW^-1^ ± standard error) for each *Vitis* genotype, and each sampling date or each plant section. Coefficient of variation (CV) corresponding to the standard error, divided by the mean value of the FDp titer was determined. Data comparison, and statistical significance evaluation, were performed using the χ^2^ and the Wilcoxon rank sum tests with software R, and R commander package (Fox, 2005). A scatterplot was generated using R, to evaluate the relationships between the relative mean FDp titers (ratio between the mean FDp titer in the genotype and the mean FDp titer in the CS from the same experiment), and the relative proportion of infected plants (ratio between the proportion of infected plants in the genotype and the proportion of infected plants in CS from the same experiment) of the different accessions inoculated in the greenhouse. Hierarchical classification analysis was performed using FactoMineR package, combining the two factors (Lê et al., 2008) to reveal the similarities between the *Vitis* accessions, with respect to FDp titers and the proportion of infected plants.

## Results

### Symptom Severity and FDp Titer in CS and M in Vineyards

**FIGURE 3:**
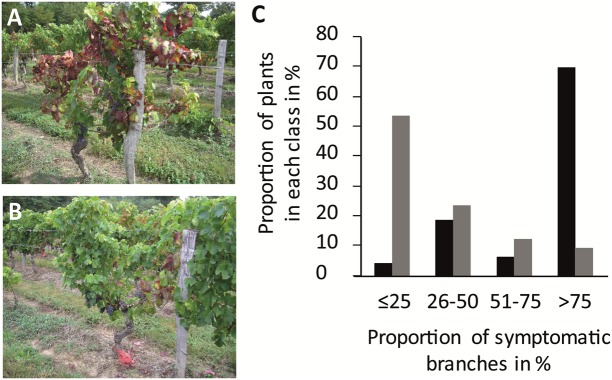
**Cabernet Sauvignon and Merlot vines (A,B)** with >75 and ≤25% symptomatic branches, respectively (photograph taken in September). **(C)** Proportion of plants with ≤25%, 26–50%, 51–75%, and >75% symptomatic branches in Cabernet Sauvignon and Merlot (black and gray bars, respectively) in the vineyard-plots of Ambès, Beychac, and Baurech (2411 symptomatic out of 6306 CS observed and 307 symptomatic out of 9268 M).

In the three vineyard plots from the municipalities of Baurech, Beychac, and Ambès, we identified 38% (2411) and 3% (307) symptomatic CS and M, respectively (**Figure 2**; Supplementary Table S1). The proportion of symptomatic M was drastically lower than CS in each whole plot and also when only the 10 rows on each side of the border between CS and M were compared (Supplementary Table S1). To better characterize the differences between these cultivars, we evaluated the number of branches showing symptoms of FD disease (**Figures 3A–C**). Four classes of plants with >0–25%, 26–50%, 51–75%, and >75% symptomatic branches were defined. Of the CS stocks, 70% showed over 75% symptomatic branches whereas 55% of M stocks showed less than 25% symptomatic branches (**Figure 3C**). Similar results were obtained on each site independently, and even when only the bordering rows were compared (Supplementary Table S1). At the end of the summer in Ambès and Baurech, and throughout a full vegetative season in Beychac, we sampled petioles on CS and M stocks (28–35 stocks/cultivar/plot), equally selected in the four classes of symptoms and randomly distributed on each plot. FDp was detected in all symptomatic samples. The *map* gene sequence of FDp collected in Beychac was identical with the one of FDCAM-05 strain (Map-FD1, Papura et al., 2009) and FDp from Ambès and Baurech was genotyped as FDPEY-05 (Map-FD2).

**FIGURE 4:**
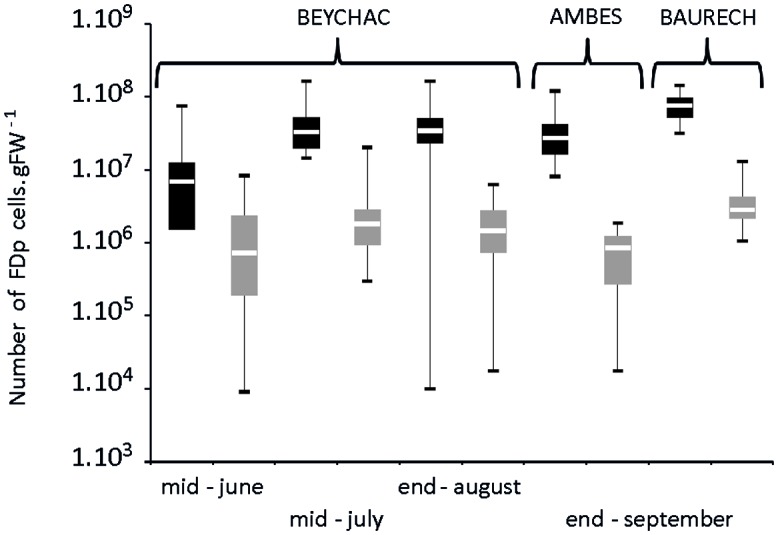
**Box plot of FDp titer in symptomatic leaves of Cabernet Sauvignon (CS) and Merlot (M) along the vegetative season in the vineyards of Beychac, Ambès, and Baurech.** The FDp titers were evaluated from 28 to 35 samples of each accession/plot in CS and M (black and gray bars, respectively). Data are statistically different between CS and M within each season, according to the Wilcoxon rank sum test (*p* < 0.001). Median (solid line), 25th and 75th percentiles (box), 10th and 90th percentiles (whisker) are presented.

In Beychac, FDp titers increased significantly between mid-June and mid-July for both cultivars and then stabilized until the end of August (**Figure 4**; Supplementary Table S1). At each time-point, FDp titers in M were inferior to those in CS with high statistical significance. The ratio between mean FDp titers in CS plants and M increased along the season from 5 times, in June, to 23 times, in autumn in the Beychac vineyard, while in Ambès and Baurech vineyards, ratios of 46 and 30 were reached, respectively (**Figure 4**). When analyses were performed on plants from the 10 rows of the border between CS and M, the ratio was 50.7 in Ambès and 30, in the site of Baurech. The plotting of FDp titers to the proportion of symptomatic branches for each M and CS plants shows that these variables are not linked (*R*^2^ < 0.02; Supplementary Figure S1). We also sampled the green arms that remained non-symptomatic on each symptomatic stock studied. Ninety-one percent and 71% were positive for FDp in M and CS, respectively. However, phytoplasma was hardly detected, at the limit of quantification for both cultivars, suggesting that non-symptomatic branches from a symptomatic plant may contain very low FDp titers, whatever the cultivar. Finally, FDp was not detected in the completely non-symptomatic stocks from the same vineyard-plots.

In conclusion, our observations demonstrate that in the same vineyards, and even in the close bordering rows, most likely subjected to similar abiotic and biotic conditions, the proportion of symptomatic plants, the proportion of symptomatic branches, and the FDp titers in symptomatic branches were lower in M than in CS.

### Symptoms and FDp Titers in Wild Rootstocks Regrowth in the Field

Wild rootstocks growing near FD-infected vine plots were sampled at the end of the summer in the Bordeaux area. Petioles were collected mainly on branches of *V. riparia* showing leaf yellowing, and on some branches of 161-49C (*V. riparia* × *V. berlandieri*) and Clinton (*V. riparia* × *V. labrusca*) that showed leaf yellowing and/or discrete rolling. Among 56 rootstocks analyzed, 37.5% were positive for FDp, with a mean titer of 2 × 10^7^ ± 6 FDp cells.gFW^-1^, which is similar to the titer observed in CS at the end of the season.

### A Protocol to Reproducibly Inoculate Vine Plants with Infectious *S. titanus* under High Confinement Greenhouse Conditions

To further characterize grapevine susceptibility, we attempted to translate what occurs in the vineyards into controlled conditions, under confinement constraints (**Figure 1**). All accessions introduced *in vitro* developed after a 6-month period. Plants became juvenile and cuttings shared very homogenous plant development. After transfer in soil, plants developed homogenously too, thus favoring insect-mediated transmission (**Figure 1**).

Through the inoculation experiments performed across 4 years, the proportion of infected *S. titanus* collected at the end of each transmission experiment varied from 83.6 to 100% and the mean FDp titers ranged from 4.5 × 10^7^ ± 9.1–3.4 × 10^8^ ± 4.8 FDp cells.insect^-1^. In such conditions, transmission efficiency in the control CS was high, with a mean proportion of 92.9% infected plants (CV 5.7%) and the first symptoms were observed 6 wpt in all experiments. For FDp titers in CS plants, the CVs were higher, ranging from 51.2 to 113.7% for each experiment and 78.3% between experiments. Inter-experiment variations were normalized by calculating the percentage of infected plants and the mean FDp titers relative to CS for each experiment [cf. Materials and Methods (M&M)]. The whole procedure lasted 24–32 weeks, and proved to be efficient on a large collection of accessions.

### Time Course of FD Multiplication and FDp Colonization in CS and M, after Transmission by *S. titanus*

**FIGURE 5:**
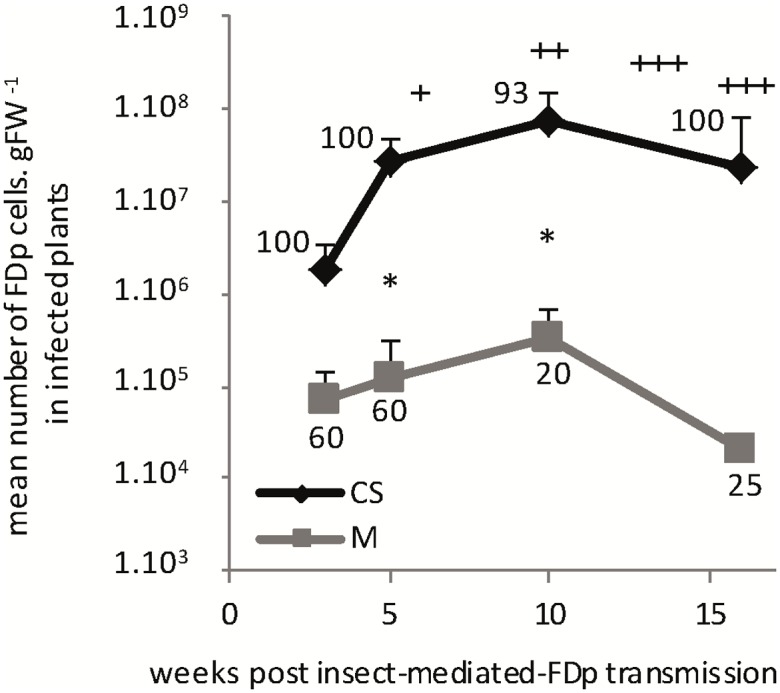
**Time course of FDp multiplication and symptoms development, after *S. titanus*-mediated transmission in Cabernet Sauvignon and Merlot.** Percentage of infected plants is indicated for each sampling time. +, discrete veins and leaf blades reddening, slow plant growth; ++, intense veins and leaf blades reddening, leaf-rolling, and plant development arrest; +++, intense reddening, leaf rolling, and drying followed by plant decay for CS. Merlot did not show any symptom. ^∗^, significant difference between CS and M values according to the Wilcoxon rank sum test (*p* < 0.05) out of 5–20 plants tested. Vertical bars indicate standard error.

For the time course experiment, infectious insects were encaged on each whole CS and M plants. The percentage of infected plants (*n* = 5–10), symptom development and mean FDp titers in the entire aerial parts were evaluated 3, 5, 10, and 16 wpt (**Figure 5**). The proportion of infected M (34.4%) was lower than CS (96.9%), as observed in the vineyards. In CS, the mean FDp titers strongly increased after transmission, with a multiplication factor of 38 between 3 and 10 wpt, and a maximum of 7 × 10^7^ ± 5.4 FDp cells.gFW^-1^ 10 wpt. The FDp titers were statistically different between all time points. The symptoms appeared between 6 and 7 wpt, close to the end of the FDp growing phase and reached their maximum intensity 12 wpt (**Figure 5**). Ten wpt, FDp titers tended to decrease, with a mean of 2.2 × 10^7^ ± 1.4 FDp cells.gFW^-1^, 16 wpt and, during this phase, plants began to decay with leaves drying and falling, with 62.5% of CS presenting dead parts of the plant. In M, FDp titers followed a similar growth curve as in CS, but with no statistical significance between sampling times. In the experimental conditions, M plants did not show any symptoms and were still growing 16 wpt (Supplementary Figure S3). Notably, FDp titers measured in M remained statistically lower than in CS. Indeed ratios between means FDp titers in CS vs M were of 224 and 241, 5 and 10 wpt, respectively, thus resembling data obtained in the vineyards.

**FIGURE 6:**
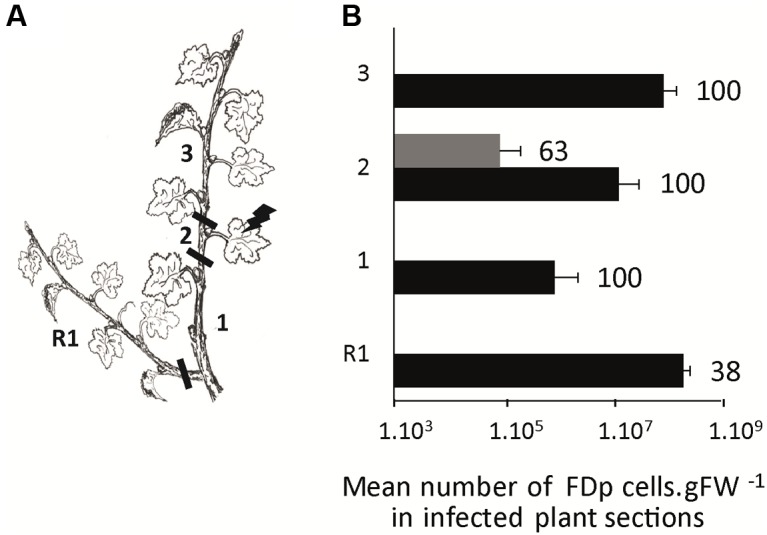
**Plant colonization by FDp after *S. titanus*-mediated transmission in Cabernet Sauvignon and Merlot.** FDp infected *S. titanus* were deposited on the leaf of section 2 **(A)**. Plants were grown 10 weeks for further development and eventually development of regrown arm (R1). The percentages of infected plants and mean FDp titer in each plant section (1–3 and R1) are shown for 10 CS and 8 Merlot plants analyzed (black and gray boxes, respectively) **(B)**. Horizontal bars indicate standard error.

To study the distribution of FDp in CS and M plants, we encaged infectious *S. titanus* on the 3rd or 4th leaf from the apex. Ten wpt, CS and M plants were cut in sections, as schematized in **Figure 6A** and FDp was quantified (**Figure 6B**). The CS sections 2, including the leaf that received the infected *S. titanus*, were 100% infected with a high mean FDp titer of 1.3 × 10^7^ ± 1.8 FDp cells.gFW^-1^ (**Figure 6B**). The other sections from the main stem were infected in the same proportion, however, with mean FDp titers 99 times higher in the apical (section 3) than in the basal parts (sections 1) (*p*-value < 0.001). The proportion of infected basal regrown arms (R1) was lower than for the other sections (38%), with a titer similar to that measured in the apex. In contrast, 63% of the M sections 2 were infected with low mean FDp titers of 8.9 × 10^4^ ± 12 FDp cells.gFW^-1^ (**Figure 6B**), while none of the other sections were infected, suggesting that FDp is almost restricted to the leaf used for transmission. Taken together, these data suggest that FDp circulates within the CS plant and multiplies preferably close to the main apical meristem or to the meristem of axillary branches initiated post-transmission, whereas the phytoplasma is arrested in M, in the site of infection.

**FIGURE 7:**
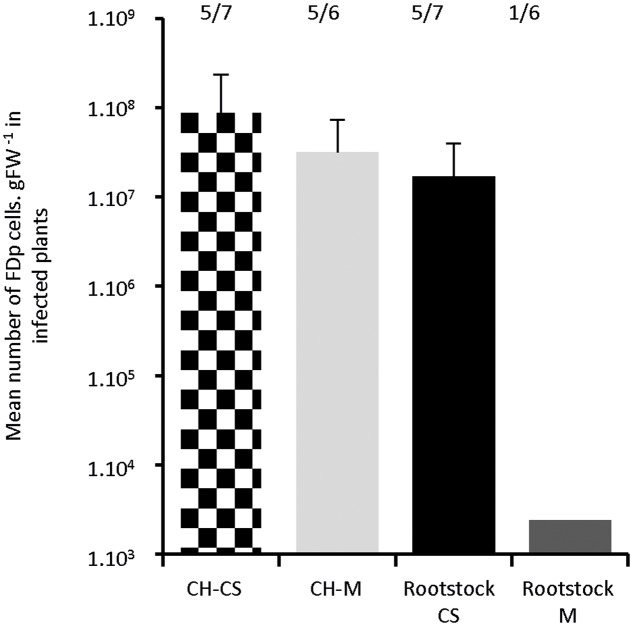
***In planta* diffusion of FDp after *S. titanus*-mediated transmission on Chardonnay scions grafted on Cabernet Sauvignon and Merlot.** Transmission of FDp by infected *S. titanus* was done on the chardonnay scion. Plants were grown for 15 weeks for further development and FDp titer was evaluated in scions of chardonnay grafted on CS, M (black square boxes and light gray bar) and in the CS and M rootstocks (black and dark gray bars, respectively) (six to seven plants each). Number of positive plants are indicated over the boxes. Vertical bars indicate standard error.

In the course of the experiments, we observed lower survival rates of *S. titanus* at the end of the transmission period on M, when compared to CS (Supplementary Table S2). This discrepancy could lead to a lower transmission efficiency within M. To elucidate whether this differential response is due to a plant–insect or FDp–plant interaction, we grafted a scion of Chardonnay, a highly susceptible cultivar, onto CS and M rootstocks. Plant tissues readily welded within 1–4 days, so that vascular tissues looked continuous. After 2 months’ further development of the grafted plants, *S. titanus* were encaged for transmission onto the Chardonnay scion solely. The Chardonnay shoots expressed symptoms 5–6 wpt for both cultivars. The proportions of infected Chardonnay scions were 71 and 83% for CS and M, respectively. The FDp titers, 15 wpt, were not statistically different in Chardonnay shoots grafted onto CS or M, suggesting that the rootstock does not influence FDp multiplication into the scion (**Figure 7**). The FDp likely diffused into the plants, through the graft, and then multiplied, reaching a high titer 15 wpt in the CS rootstocks, with a mean of 1.7 × 10^7^ ± 2.2 FDp cells.gFW^-1^ and 100% infection rate. In contrast, a very low titer of 2.4 × 10^3^ was found in a single M rootstock, whereas the other five remained FDp-free. So, although we do not exclude an influence of plant cultivar on the transmission step by *S. titanus*, here the FDp–plant interaction *per se* can explain the lower susceptibility of M, when compared to CS.

### A Wide Range of Susceptibility to FD in *V. vinifera* Cultivars, Rootstocks, and Wild *Vitis* Species

**FIGURE 8:**
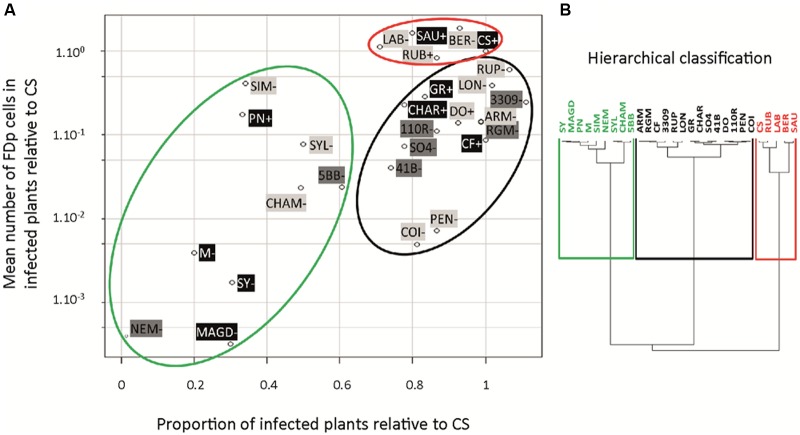
***Vitis vinifera* cultivars, rootstocks, and wild *Vitis* sp. susceptibility to FD disease.** *S. titanus*-mediated transmissions of FDp to plants were performed under controlled greenhouse conditions. Ten weeks later, the proportion of infected plants was evaluated and expressed relative to Cabernet Sauvignon. The mean FDp titer was evaluated, after qPCR (log scale) and expressed relative to Cabernet Sauvignon. These two sets of data were plotted **(A)**. The hierarchical clustering shows the most susceptible genotypes in red, intermediate in black, and less susceptible in green **(B)**. *V. vinifera*, rootstock, and wild *Vitis* species (black, dark gray, and light gray, respectively; - or + indicate the absence/presence of symptoms before 10 wpt; data collected from 9 to 20 plants of each accession).

A collection of 28 *Vitis* accessions was introduced and cultivated *in vitro* (**Table 1**). Accessions were free of virus Grapevine virus A (GVA), grapevine leafroll-associated virus (GLRaV) 1–3, and grapevine fanleaf virus (GFLV), except CS 337, which was infected with GLRaV 2 (data not shown). The percentage of infected plants, symptom development, and mean FDp titers in the entire aerial parts were evaluated 5 and 10 wpt. A total of seven transmission experiments were performed (Supplementary Table S2), with the responses of the control CS being used as a reference, in each experiment. The scatterplot (**Figure 8**) integrated for each accession the mean relative FDp titer in infected plants and the proportion of infected plants, relative to CS. On the basis of these values, a hierarchical clustering suggested three categories (**Figure 8B**): (1) accessions with high FDp titers and high proportion of infected plants, (2) accessions with intermediate FDp titers and high proportion of infected plants, and (3) accessions with intermediate to low FDp titers and low proportion of infected plants (red, black, and green circles, respectively in **Figure 8A**). *V. vinifera* cultivars were distributed throughout these three categories. Sauvignon was classified as highly susceptible with FDp titers and proportion of infected plants not statistically different from those measured for CS (Supplementary Table S2). On the opposite, M, Syrah, and Magdeleine were classified as poorly susceptible, showing FDp titers and proportion of infected plants much lower than in CS, with high statistical significance. Pinot Noir, although included in the same hierarchical cluster than M, showed a more susceptible phenotype, with low proportion of infected plants but intermediate FDp titers. Finally, Grenache, Chardonnay, and Cabernet Franc were classified as intermediate, with intermediate FDp titers, but high proportion of infected plants. The cultivars with high or intermediate FDp titers showed symptoms (**Figure 8**; Supplementary Figure S3) while those with low titers did not show any symptoms 10 wpt (**Figure 8**).

For the rootstocks, with the exception of 5BB and Nemadex, which were classified as poorly susceptible, all accessions were grouped in the category of “intermediate susceptibility.” They showed high proportions of infected plants and intermediate relative FDp titers, ranging from 0.04 for 41B to 0.25 for 3309C. The 5BB was less susceptible, with significantly lower proportion of infected plants and FDp titers. Finally, in Nemadex, a complex hybrid with a *Muscadinia rotundifolia* genetic background, FDp was barely detectable and plants did not show any symptoms (Supplementary Figure S2). Interestingly, none of the rootstocks exhibited FD symptoms in our experimental conditions (**Figure 8**), even those having the highest FDp titers.

The 12 wild *Vitis* species analyzed were distributed among the three categories, with diverse susceptibilities. As observed for the rootstocks, they could be infected and have high FDp titers, without developing any symptoms 10 wpt, except for *Vitis doaniana* and *Vitis rubra* which showed yellowing and rolling of the leaves 7–9 wpt. *V. berlandieri*, *Vitis rupestris*, and *V. riparia* (RGM), the species most used decades ago in rootstock breeding schemes to produce the main rootstocks used today, showed high proportions of infected plants and intermediate to high FDp titers. However, none showed symptoms 10 wpt. A high proportion of *Vitis pentagona* and *Vitis coignetiae* were infected, but the FDp titers were low. Conversely, low proportions of infected plants, but with high FDp titers were obtained with *Vitis simpsonii*. These observations suggest that the efficiency of transmission, on one hand, and the FDp titers, on the other hand, are likely associated with distinct plant traits. The same stands true for the FDp titers and symptom development, which are unlinked in all rootstocks studied.

## Discussion

In the three vine plots studied, we showed that the percentage of infected plants and the percentage of symptomatic branches on each stock were lower for M than for CS. These results are in accordance with observations collected by the services in charge of the disease’s survey, on a larger scale, in the Bordeaux vineyards (Vergnes, personal communication) and with former observations (compiled in Galet, 1982 and in Boudon-Padieu, 1996). We also showed that FDp titers increased along the summer in symptomatic shoots of both cultivars. Such seasonal trends were observed in Slovenia, with Blaufrankisch and Refosk cultivars (Prezelj et al., 2013) and in Italy, with Barbera and Nebbiolo cultivars (Roggia et al., 2014). A direct correlation between FDp titer in the plant and its acquisition efficiency by the insect has been shown (Galetto et al., 2014). Therefore, elucidating whether FDp titer is linked to symptom development is a key issue for the mandatory surveys. Vineyard’s data suggest that within a given cultivar, the relationship between symptom severity and FDp titer is not established for CS and M, nor for Barbera and Nebbiolo, in three sampling periods out of four (Roggia et al., 2014). However, whatever the sampling period, the site of sampling and the FDp genotype infecting the plot, titers were always lower in M than in CS. Other comparative studies conducted in the vineyards, though on a smaller scale, reached the same conclusions: Barbera is highly susceptible with high FDp titers and severe symptoms, while the poorly susceptible Nebbiolo shows low FDp titers and almost lacked symptoms (Roggia et al., 2014). Furthermore, on the basis of the nine different cultivars infected under controlled conditions, which were also observed in large number in vineyards (for review Galet, 1982; Boudon-Padieu, 1996), we infer that *V. vinifera* cultivars expressing severe symptoms do also have high FDp titers (CS, Sauvignon, Grenache, Chardonnay) while in those poorly expressing symptoms the phytoplasma is poorly multiplying (M, Syrah, Magdeleine). Although drawn from low number of plants and from a single vine plot, recent results suggest possible exception, with the cultivar Arneis which is described as highly susceptible in the field but shows low FDp titers (Galetto et al., 2016).

Although vineyard experiments allow for a true FDp-vine’s interactions analysis, numerous factors are in play that undermine the conclusions to be drawn. For example, the temperature influences the multiplication of FDp in plants (Salar et al., 2013) and there is a direct correlation between insect population and percentage of infected plants (Morone et al., 2007) and both factors can vary between observation sites. The number of plants is often low, as mandatory actions lead to uprooting of symptomatic plants. In this respect, our comparative study on thousands of CS and M plants relies on three independent FD-infected vineyards, with both cultivars having the same age, grafted with the same rootstock and planted adjacent to each other. Therefore, our data are rather conclusive in saying that CS is highly susceptible to FD, whereas M can be considered as poorly susceptible, namely as it hampers the diffusion of FDp within the plant, as well as its multiplication. Such experimental conditions are rather rare, though. Furthermore, vineyards are not the place for evaluating the susceptibility of diverse *Vitis* accessions, as they are not planted, thus reinforcing the interest of experimental FD transmission in controlled conditions.

Diverse experimental inoculation protocols have been developed for evaluating the susceptibility of perennial host plants to different vector-borne diseases. However, most of these do not use natural transmission by the vector. For example, *Xylella fastidiosa* was mechanically (needle) inoculated in order to evaluate the relative susceptibility of different grapevine cultivars (Rashed et al., 2013). The phytoplasmas *Candidatus* Phytoplasma (*Ca.* P.) prunorum and *Ca.* P. mali, were transmitted to young *Prunus* sp. and *Malus* sp. rootstocks by *in vitro* grafting (Jarausch et al., 1999; Seemüller et al., 2007). In the case of phytoplasma, mechanical inoculation of plants never succeeded, and grafting of FDp-infected grapevines led to low transmission efficiency. The highest rate reported (51.3%) was obtained by auto-grafting from green shoots of the cultivar Plovdina, which is susceptible to FD (Kuzmanović et al., 2011). The true natural transmission, which covers plant–insect interactions, bacterium–insect, as well as plant–bacterium interactions will remain the most informative, as illustrated in other pathosystems. Thus, feeding of the vector *Aphis gossypii* triggers a localized hypersensitive response of melon genotypes carrying the *vat* gene, with cell death and callose deposition, which restrains the propagation of viruses to the feeding point (Villada et al., 2009).

We therefore developed a protocol to reproducibly obtain vine plants infected by *S. titanus.* The feeding of *S. titanus* on FDp-infected broad beans insured high acquisition rates as already described (Bressan et al., 2005) and also permitted high transmission rates on the susceptible CS. Our multi-step protocol allows for the production of reproducible data on a quantitative level, resembling at most, the natural conditions. But what takes months in vineyards (symptoms development and FDp multiplication) took weeks here.

After localized transmission on one leaf of the mid-apical part, the phytoplasma multiply, circulate within CS, and then colonize the entire plant, with higher titers in the apical than in basal parts as observed with Onion yellows and *Chrysanthemum* yellows phytoplasmas in *Chrysanthemum carinatum* (Wei et al., 2004; Saracco et al., 2005). In M, FDp was only detected in the section used for transmission, throughout weeks, suggesting that the plant is arresting the phytoplasma at its entry point where, moreover, the FDp titers remain low. Arresting of the phytoplasma in M was also observed after inoculation of a grafted Chardonnay shoot. This specific response, first described in grapevine, reflects a plant–phytoplasma interaction, unbiased by plant–insect interactions. This is opening for refined biochemical and molecular analysis of specific cultivar/phytoplasma interactions as preliminary studied in Nebbiolo and Barbera (Margaria and Palmano, 2011a,b).

Results of greenhouse inoculations on 28 *Vitis* sp. accessions showed a wide range of susceptibility. When combined with extended field observations, our study allows to infer that all *V. vinifera* develop symptoms in response to FD, though with different severities, which correlate with different FDp titers, suggesting the presence of quantitative traits in this germplasm. Interestingly, the mother cultivar of M, the Magdeleine (Boursiquot et al., 2009) was poorly susceptible whereas M’s “step brother,” CS and its parent Sauvignon were both highly susceptible. Cabernet Franc, the common parent of M and CS was ranked as intermediate. This reinforces our hypothesis of a genetic determinism for quantitative plant responses. A poor insect survival was observed at the end of the transmission period for the three less susceptible cultivars M, Syrah, and Magdeleine (Supplementary Table S2). The low survival could be enhanced by the presence of the phytoplasma in their body, which also alter their fitness, as demonstrated in Bressan et al. (2005). It suggests that *S. titanus* might have difficulties in correctly feeding and inoculating FDp in the phloem of these cultivars, and that the first barrier to FD spreading in the plant is already at this particular step. Our data reinforce the importance of using natural transmission involving all partners for precise investigations.

With respect to the rootstocks, in all but Nemadex, insect survival and percentage of infected plants were quite high. The same was true for the North-American wild *Vitis* sp. (*V. riparia*, *V. berlandieri*, and *V. rupestris*), which were originally hybridized to obtain these rootstocks. As wild North-American *Vitis* sp. are the original hosts of *S. titanus* (for review Chuche and Thiéry, 2014), the insect might be well adapted to feed in the phloem of such species and thus, efficiently transmit the phytoplasma. Also, we observed high populations of *S. titanus* on the wild rootstocks regrowth, which were collected in the field for the study (data not shown), and high populations have also been recorded in different European regions (Lessio et al., 2014; Strauss et al., 2014), which strengthens the hypothesis that the insects feed and survive well on these plants. The rootstocks and the original *Vitis* sp. exhibited intermediate to high FDp titers, but did not express any symptoms in greenhouse. This is in accordance with former field observations and experimental inoculations by grafting, which showed that some infected rootstock varieties, such as 41B or 5BB, did not express symptoms or, like 3309C, displayed attenuated ones (some yellowing, incomplete lignification, and growth delay; Caudwell et al., 1994a,b; Boudon-Padieu, 1996; Borgo et al., 2009). This is of particular concern for mother plant rootstocks, which in Europe, are not surveyed for the presence of FDp and can constitute a source of FDp contamination by grafting. The same phenotype was observed for the wild rootstocks regrowth which are not subjected to the mandatory control measures. This is also preoccupying as it has been demonstrated that *S. titanus* can migrate from the rootstocks to the vineyards nearby (Lessio et al., 2014). Therefore, they can constitute reservoirs of vectors and FDp for healthy vineyards or in the process of sanitation. In contrast, we could hardly detect phytoplasma in the Nemadex rootstock, suggesting a degree of immunity to FD. To rule out this issue, we had to dramatically increase the “disease pressure,” namely by multiplying the number of high-FDp-containing insects used for transmission. Under these circumstances, barely comparable to natural vineyards conditions, we uncovered infected plants, however, FDp titers still remained as low as in M (data not shown). In further studies, it will be interesting to evaluate the susceptibility of *M. rotundifolia*, which constitutes the genetic background of the poorly susceptible Nemadex rootstock.

In plants, wild accessions are often a germplasm resource for breeding of disease resistance traits. Thus, in *Vitis*, *V. labrusca* was a source for mildew resistance (Cadle-Davidson, 2008), and various American *Vitis* (*V. berlandieri*, *V. riparia*, *V. rupestris*) were introduced in Europe to fight against phylloxera in the 19th century. Interestingly, *V. simpsonii* showed low proportions of infected plants, with low FDp titers, whereas *V. coignetiae* and *V. pentagona* showed high proportions of infected plants with low FDp titers, suggesting unlinked traits for the response to transmission and phytoplasma multiplication, respectively. However, here, none of the 13 wild species tested appeared as a source of resistance.

As a conclusion, our data suggest that to cope with the disease, genetic traits of interest can rather be found in the *V. vinifera* germplasm. Linked or not, they are involved in insect-mediated FDp transmission, multiplication, circulation, and symptom development. Our transmission assays under controlled conditions gave rise to data coherent with field observations published so far and offer new ways for refining genetic and molecular characteristics of vine responses to FD, including means to cope with the disease in the vineyards.

## Author Contributions

SE, XF, JM, and SM-M conceived and planned the study. CJ, FL, DK, MP, DD, PS, FR, CH, JM, SE, and SM-M performed experimental procedures and collected data. LB helped in the *Vitis* identification and gave advices. SE, JM, XF, and SM-M wrote the manuscript.

## Conflict of Interest Statement

The authors declare that the research was conducted in the absence of any commercial or financial relationships that could be construed as a potential conflict of interest.
