# Supplementary material for: Contrasting Susceptibilities to Flavescence Dorée in Vitis vinifera, Rootstocks and Wild Vitis Species
Source: Front Plant Sci. 2016 Nov 29;7:1762. doi: 10.3389/fpls.2016.01762 (PMC5126068; doi:10.3389/fpls.2016.01762)
Supplement: Supplementary file 1 [file Data_Sheet_1.DOCX]

Supplementary Material

**Contrasting susceptibilities to Flavescence dorée in *Vitis* *vinifera*, rootstocks and wild *Vitis* species**

S. Eveillard^1*^, C. Jollard^1^, F. Labroussaa^1^, D. Khalil^1^, M. Perrin^2^, D. Desqué^1^, P. Salar^1^, F. Razan^1^, C. Hévin^3^, L. Bordenave^3^, X. Foissac^1^, J. E. Masson^2^, S. Malembic-Maher^1^

* **Correspondence:** Sandrine.Eveillard@bordeaux.inra.fr

**Supplementary notes on q-PCR test based on the *tuf* gene**

**Phytoplasma strains and isolates**

Phytoplasma strains of the 16SrV group: FD-CAM05 (Map-FD1), FD-PEY05 (Map-FD1), FD-Loza (Map-FD3), Rubus Stunt (RuS), Elm Yellows (EY1), EY-17-49 (PGYA) and EY38 (PGY C) (Arnaud *et al.*, 2007) were used to test the polyvalence of the real-time PCR quantification of FDp. Phytoplasma strains from other taxonomic groups which have already been described infecting grapevine worldwide: Aster Yellows Whitcomb 16SrI, Peach western X 16SrIII, *Ca*. P. australiense 16SrXI, GGY 16SrXII were used to test the specificity of the real-time PCR. All phytoplasma strains were maintained on periwinkle in our greenhouse collection in Bordeaux except for the FD-Loza isolate, which was kept as total DNA from an infected grapevine from Serbia.

**Quantitative real-time PCR conditions**

Primers for real-time PCR were designed on the basis of the *tuf* gene sequences alignment of reference isolates representative of the 16SrV group and other taxonomic groups available in databases AM939565 (Malembic-Maher *et al.*, 2008); FN561871 to FN561875; FN561877; FN561879 to FN561881; FN561884 to FN561887 (Malembic-Maher *et al.*, 2011); EF635115 to EF635120; (Šeruga Musić *et al.*, 2008), DQ096804 (Andersen et *al.*, 2005, unpublished) and AJ271321 (Marcone *et al.*, 2000). Determined primers 3Fbl (5’-TGAAGATCCAGTACGTGATTTAGAC-3’) and 3Rl (5’-TTTTAGTTTCTTTAATACCTATGATTTC-3’) are positioned at base pairs 573–597 and 702–730, respectively, on sequence AM939565 and led to the amplification of a 158 bp fragment from all FDp strains.

Different amount of nucleic acids extracted from naturally infected CS and M (from 100 to 500 ng) were tested to determine the optimum amount beyond which there is no correlation between phytoplasma titer and the amount of nucleic acid template. In parallel, different amounts of *Vitis* nucleic acids from healthy plants (100 to 500 ng) were mixed with standard plasmids to confirm the optimum amount of nucleic acids to avoid PCR inhibition.

The standard corresponded to a fragment of the *tuf* gene sequence amplified from total nucleic acids of FD-PEY05 infected broad bean, using primers FDTUF-F1/FDTUF-R1 and FDTUF-F2/FDTUF-R2 as described in Malembic-Maher *et al.*, 2011 and cloned in the pGEM-Teasy (Promega) following manufacturer recommendations.

Different amount of nucleic acids extracted from naturally infected CS and M (from 100 to 500 ng) were tested to determine the optimum for sensitive detection and linear quantification of FDp.

Thereafter, real-time PCR assays were conducted with 200 ng nucleic acids from the *Vitis* samples as template using LightCycler ® 480 SYBR Green I Master (Roche) and primers at 375 nM (3Fbl) and 750 nM (3Rl). Amplification was performed in 96-well plates ([LightCycler® 480 Multiwell Plate 96](http://www.roche-applied-science.com/shop/en/fr/products/lightcycler14301-480-multiwell-plate-96)) on a 480 LightCycler Real-Time PCR detection system with the following cycles: 1 cycle at 95 °C for 15 minutes, and 45 cycles of 94 °C for 15 sec, 60 °C for 30 sec and 66 °C for 30 sec. A melting-curve temperature profile was obtained after 95 °C for 10 sec, 66 °C for 10 sec and heating to 95 °C with a ramp rate of 0.11 °C/sec followed by a cooling to 50 °C at 2.2 °C/sec. Standard curves for the absolute quantification of FDp were obtained by testing serial dilutions of a plasmid standard from 1x10^8^ to 1x10^1^ copies mixed with 200 ng total nucleic acids of healthy grapevine. Each sample was deposited in duplicate and the mean number of *tuf* gene copies between both duplicate was calculated. Data acquisition and analysis were handled by the LightCycler® 480 software release 1.5.0 (Roche), which automatically calculates the number of *tuf* gene copies in each sample by comparison with the standard range. Finally, as the FDp complete sequence genome (Carle *et al.*, 2011) showed that it contains just a single copy of *tuf* gene, all data are presented as a number of FDp cells/g Fresh Weight (FW) of plant material.

Intra- and inter-plate reproducibility and PCR sensitivity were evaluated by testing 6–7 replicates of plasmid dilutions from 1x10^8^ to 1x10^1^ plasmid copies mixed with 200 ng nucleic acids from healthy grapevine, repeated on three different plates. The coefficient of variation (CV) corresponding to the standard error divided by the mean value of the FDp titre was determined for each standard dilution both for intra- and inter-plate reproducibility. Reproducibility was also tested on 12 vineyard FD-infected samples (CS and M) in quadruplicate and repeated on two plates.

**qPCR performances**

When 100 to 300 ng nucleic acids extracted from infected CS or M were used as template to determine phytoplasma titers, amplification resulted in linear regression line with a high correlation coefficient (0.95<R^2^<0.99) (Fig S4-A). In parallel, amplifications of the *tuf* gene fragment from serial dilutions of the standard plasmid mixed with 200 ng of healthy grapevine nucleic acids resulted in linear regression lines with a high correlation coefficient (0.98<R^2^≤0.99) (Fig. S4-B). PCR efficiencies varied between 82% and 99.5%, and the detection limit of the standard was reliable for as low as 10 copies per assay. An amplification curve and a unique melting pic at 76.7 °C (±0.3 °C) was observed for the standard plasmid, for all the samples infected with phytoplasma strains of the 16SrV group, and for all the FDp-infected grapevine and insect samples from field and greenhouse (Fig S4-C). No amplification curve was observed for water, healthy plants and insects and for samples infected with phytoplasma strains from other taxonomic groups. Both intra- and inter-plate CVs were under 15% for greater than or equal to 10^3^ standard plasmid copies per well. The CVs were less than 35% for 100 copies per well but reached up to 70% for 10 copies per well. The 12 tested CS and M samples (which ranged from 1.93x10^2^ to 6.44x10^5^ copies per well) were tested and calculated CVs were similar as for the plasmid (data not shown).

It is known that nucleic acid extracts from grapevine leaves and petioles are rich in flavonoids and polyphenols, often bound to DNA and RNA, which therefore hamper PCR reactions. The extraction conditions (see methods) proved to produce high-quality nucleic acids suitable for PCR tests. The PCR conditions for the 158bp *tuf* gene fragment were thus optimized with high PCR efficiency and sensitivities down to 10 copies in the presence of grapevine total nucleic acids. The test was specific for phytoplasmas of the 16S rDNA group V and presented a high reproducibility down to 1x10^2^ copies, comparable to other studies developing real-time qPCR for the quantification of phytoplasmas in plant samples (Jarausch *et al.*, 2004; Monti *et al.*, 2013). Hence, the test developed here allows specific, sensitive and reproducible detection and quantification of FDp present in plant samples either originating from vineyard or from controlled transmissions conducted with the FD-PEY05 strain.

**References**

**Arnaud G, Malembic-Maher S, Salar P, Bonnet P, Maixner M, Marcone C, Boudon-Padieu E, Foissac X**. 2007. Multilocus Sequence Typing Confirms the Close Genetic Interrelatedness of Three Distinct Flavescence Dor?e Phytoplasma Strain Clusters and Group 16SrV Phytoplasmas Infecting Grapevine and Alder in Europe. Applied and Environmental Microbiology **73**, 4001–4010.

**Carle P, Malembic-Maher S, Arricau-Bouvery N, Desqué D, Eveillard S, Carrère S, Foissac X**. 2011. Flavescence dorée phytoplasma genome: a metabolism oriented towards glycolysis and protein degradation. Bull Insectol (Supplement) **64**, S13–S14.

**Jarausch W, Peccerella T, Schwind N, Jarausch B, Krczal G**. 2004. Establishment of a quantitative real-time PCR assay for the quantification of apple proliferation phytoplasmas in plants and insects. Acta Horticulturae, 415–420.

**Malembic-Maher S, Constable F, Cimerman A, Arnaud G, Carle P, Foissac X, Boudon-Padieu E**. 2008. A chromosome map of the Flavescence doree phytoplasma. Microbiology **154**, 1454–1463.

**Malembic-Maher S, Salar P, Filippin L, Carle P, Angelini E, Foissac X**. 2011. Genetic diversity of European phytoplasmas of the 16SrV taxonomic group and proposal of ‘Candidatus Phytoplasma rubi’. International Journal of Systematic and Evolutionary Microbiology **61**, 2129–2134.

**Marcone C, Lee IM, Davis RE, Ragozzino A, Seemüller E**. 2000. Classification of aster yellows-group phytoplasmas based on combined analyses of rRNA and tuf gene sequences. International Journal of Systematic and Evolutionary Microbiology **50**, 1703–1713.

**Monti M, Martini M, Tedeschi R**. 2013. EvaGreen Real-time PCR protocol for specific ‘Candidatus Phytoplasma mali’ detection and quantification in insects. Molecular and Cellular Probes **27**, 129–136.

**Šeruga Musić M, Krajačić M, Škorić D**. 2008. The use of SSCP analysis in the assessment of phytoplasma gene variability. Journal of Microbiological Methods **73**, 69–72.

a. Total number in the plot

b. Chi2 value indicate that the number of symptomatic plants is influenced by the cultivar

c. Percentages of symptomatic plants showing ≤25 %, 26–50 %, 51–75 %, >75 % of symptomatic branches

d. Chi2 value indicates that symptom severity is influenced by the cultivar

e. P-value indicates significant difference between the values of FDp titers in CS and in M according to the Wilcoxon rank sum test

**Table S1.** Incidence of the disease, symptom severity and FDp titers for Cabernet Sauvignon (CS) and Merlot (M) growing side by side in the FD-infected plots of Ambès, Baurech and Beychac.

a. Chi2 value indicates that number of infected plants or insect survival is influenced by accession in comparison with CS from the same experiment (* < 0,05 ; ** < 0,01 ; *** < 0,001)

b. Proportion of infected plants relative to CS from the same experiment

c. P-value indicates statistical significance between the values of phytoplasma titers in the genotype and in the CS from the same experiment according to the Wilcoxon rank sum test (* < 0,05 ; ** < 0,01 ; *** < 0,001)

d. Percentage of insect survival after transmission period

**Table S2.** Data collected after FDp-inoculation by *S. titanus* to *V. vinifera* cultivars, rootstock hybrids and wild *Vitis* species.


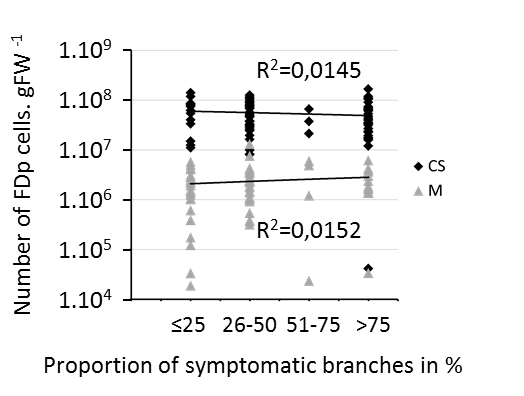


**Figure S1.** Relation between FDp titers and symptom severity in vineyards


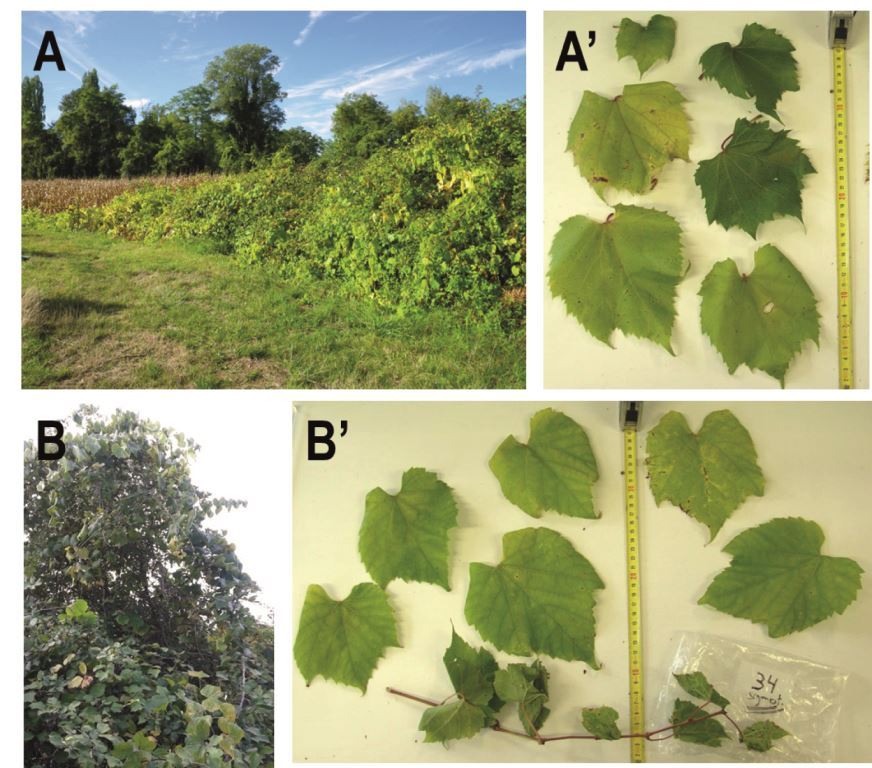


**Figure S2.** FDp-infected wild-gone rootstocks (*Vitis spp.* hybrids) showing yellowing only (A, A’) or showing yellowing and discrete rolling of the leaves (B, B’).


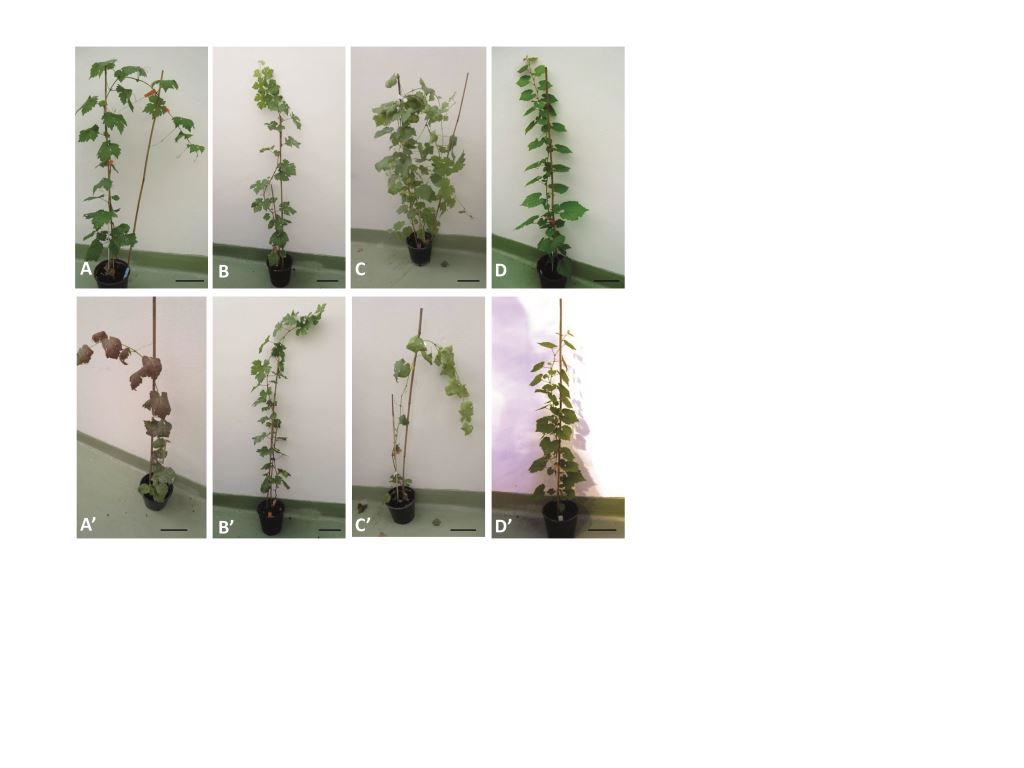


**Figure S3.** Control (top) and infected (bottom) Cabernet Sauvignon (A, A’), Merlot (B, B’), Sauvignon (C, C’) and Nemadex (D, D’), 10 weeks after *S. titanus-*mediated FDp transmission. bar=10 cm.


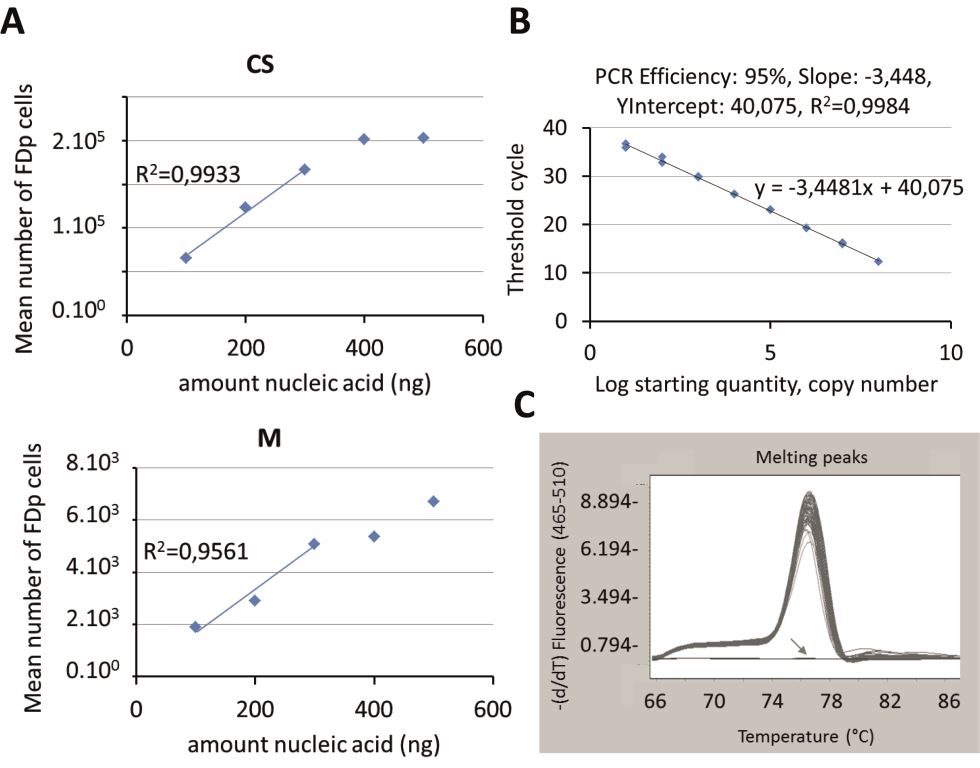


**Figure S4.** Performances of the quantitative real-time PCR based on *tuf* gene for FDp quantification. (A) Amplification curves with 100 to 500 ng infected CS or M nucleic acids as template. (B) Example of calibration curve obtained by dilution series of the standard plasmid in 200 ng nucleic acid extracts of healthy grapevine. (C) Melting curves of the amplicons obtained with primers 3Fbl and 3Rl from FDp-infected grapevines. The arrow indicates the melting curve for healthy grapevine.
